# RinPy, a Python Package for Residue Interaction Network Model to Analyze Protein Structures and Predict Ligand Binding Sites

**DOI:** 10.1021/acs.jcim.6c00004

**Published:** 2026-04-06

**Authors:** Zehra Sarica, Fethiye Aylin Sungur, Ozge Kurkcuoglu

**Affiliations:** † Computational Science and Engineering Division, Informatics Institute, 52971 Istanbul Technical University, Istanbul 34469, Türkiye; ‡ Department of Chemical Engineering, Istanbul Technical University, Istanbul 34469, Türkiye

## Abstract

The residue interaction network (RIN) model has been a useful approach for identifying functional residues and ligand-binding sites in different protein structures based on their contact topology. Here, we present RinPy, a pip-installable Python package, and a graphical user interface (GUI) that depends on a RIN model. RinPy is designed for constructing, visualizing, and analyzing RINs for single or multiple protein input structures as well as molecular dynamics trajectories, allowing the calculation of three centrality measures, namely degree, closeness, and betweenness of the residues. The nodes with the highest betweenness scores are used to suggest putative allosteric sites, and the graph spectral analysis provides hinge regions linking close-neighboring clusters. The program is enhanced using a multiprocessing module to enable efficient analysis of large protein structures and employing user-defined parameters necessary for network analysis. The package supports the integration of nucleotides, cofactors, small molecules, water, and ions directly into the network analysis, which may be highly beneficial to understand the functional interactions in protein complexes. The program consists of numerous modules producing multiple outputs, including interactive visualizations to facilitate interpretation of the results. It also provides comparative network analysis to quantify how perturbations, such as ligand binding or mutations, may alter the contact topology of protein complexes. RinPy is implemented to a data set of monomeric KRAS and KRAS–SOS1 complexes as a case study, demonstrating its ability to identify known functional and allosteric site residues and highlighting its rapid calculation and analysis of RINs. The RinPy package is compatible with Windows, macOS, and Linux operating systems. RinPy is an open-source Python package distributed via the Python Package Index (PyPI) under the name *rinpy*, with its source code available on GitHub. The RinPy GUI is also available as a standalone executable for Windows.

## Graphical abstract

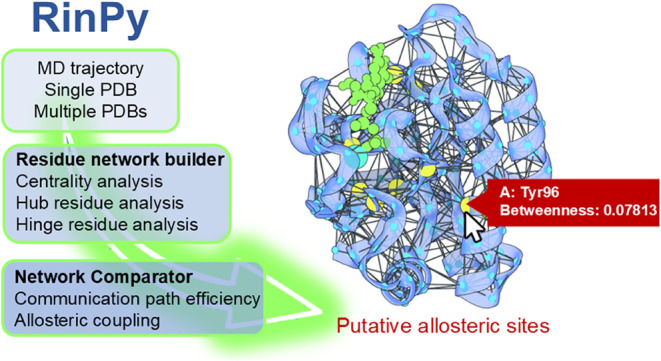

## Introduction

Allosteric regulation in protein structures has been recognized as playing a key role in many biological processes, such as signal transduction, molecular function, transcriptional regulation, and metabolism.^1–5^ Allosteric mechanisms of the proteins and their complexes can be exploited to modulate their functional dynamics in the active sites, either enhancing activation or promoting inhibition, by perturbing the allosteric sites through binding of a ligand. In line with this, allosteric drugs have become the focus of medicinal chemistry since they can selectively bind to the allosteric sites usually populated with unconserved amino acids, in contrast to orthosteric sites, which are often well conserved among species.^6^ Between 2018 and 2022, the FDA approved 24 allosteric drugs, representing ∼6% of the total number of drugs approved during this period, highlighting the therapeutic relevance of allosteric modulators,^7^ but at the same time, the difficulty of developing such compounds. Furthermore, these alternative binding sites can be particularly useful for targeting proteins that are typically regarded as ″undruggable″ because they lack clearly defined pockets and for tackling drug resistance mechanisms. The identification and characterization of allosteric sites on protein structures has grown in importance, where computer-aided drug design techniques provide a structural rationale for the discovery of highly selective and functionally varied therapeutic agents and speed up the allosteric modulator discovery process.^5^

There are various computational techniques available as Web servers or programs to predict allosteric pockets/binding sites of proteins in the literature,^8,9^ using machine learning, such as AlloPred,^10^ AllositePro,^11^ Allosite,^12^ and PASSer;^13^ and solvent mapping, such as SILCS,^14^ DruGUI,^15^ and FTMap.^16,17^ In addition, numerous computational tools have been developed for protein contact network analysis from molecular dynamics (MD) simulations or a single protein structure, including MD-TASK,^18^ NAPS,^19^ gRINN,^20^ RING 3.0,^21^ RIP-MD,^22^ RINerator,^23^ RINalyzer,^23^ and RING 2.0.^24^ Other tools are also available, such as SPACER,^25^ PARS,^26^ Allosigma,^27^ Arpeggio,^28^ and PDBe-Arpeggio.

Here, we present RinPy, the standalone Python package with a graphical user interface (GUI) that can generate a residue interaction network (RIN) of a protein complex to identify its potential allosteric regions, previously employed for the bacterial ribosome,^29^ class A GPCRs,^30^ SOS1 and KRAS–SOS1 complexes,^31^ main protease of SARS-CoV-2,^32,33^ *S. aureus* pyruvate kinase,^34^ and other glycolytic enzymes.^35^ This model has a statistically high prediction ability to detect known allosteric/orthosteric binding sites, even using a low number of structural data and/or poor resolution, as shown for GPCRs.^30,36^ The Python package is compatible with all major platforms where Python is supported, including Windows, macOS, and Linux environments. In addition, the RinPy GUI is provided as a standalone executable for Windows. In its implementation, RinPy allows the upload of input structures either by (i) fetching from the Protein Data Bank^37^ (rcsb.org) by using PDB IDs, (ii) selecting the structural data stored in PDB format from a folder in the workspace, or (iii) selecting a single PDB file containing MD trajectory snapshots. The program models each input structure as a separate RIN, where it is possible to incorporate heteroatoms, such as nucleotides, cofactors, ligands, solvents, and crystal waters into the network. Three centrality measures of the nodes, i.e., degree, closeness and betweenness, indicate functional importance of the corresponding nodes (residues/nucleotides) in the protein complex.^29^ RinPy performs the calculation of these measures with specified threshold values to filter the nodes with high centrality values. The program can generate a 3-D structure of the network highlighting the residues with high centrality scores based on the available structural data. One of the key features that distinguishes RinPy is the capacity to identify high-scored residues that are commonly observed within any set of related proteins. Thus, it can provide insight into functionally key residues, interactions, and identification of critical residues that could be suggested for mutation studies. In addition, the generated RIN can be subjected to graph spectral analysis to partition the network into close-neighboring node clusters that correspond to dynamic domains separated by flexible hinge regions, highly critical in protein functional dynamics. The program generates an output to indicate the nodes (residues/nucleotides) at the hinge regions. Residues with high betweenness values located at the hinge regions are suggested as ligand binding sites.^30,36,38^ RinPy also provides a comparative analysis between two networks, such as apo and ligand-bound structures, and evaluates how the centrality measures and the efficiency of the communication may change upon perturbation, and how a perturbation may propagate in the structure. The multiprocessing calculation was performed while designing the RinPy package to ensure computational efficiency.

To validate and evaluate the performance of the implemented algorithm, *Homo sapiens* KRAS monomer structures and KRAS–SOS1 complexes available in the Protein Data Bank were investigated as case studies. Accordingly, we successfully validated the known allosteric sites for the given case studies that had been predicted in previous studies, thus confirming their functional significance.

## Materials and Methods

### Theoretical Background

The residue interaction network (RIN) model is based on the contact topology of the protein complex structure and describes it as a network of nodes interconnected by weighted edges. Each residue, nucleotide, or ligand is represented by a node placed at the average coordinate of its atomic coordinates. The weight of the edge between two nodes is calculated using the local interaction strength *a*_*ij*_ between two residues as defined in eq 1,

1 $$a_{ij}=\frac{N_{ij}}{\sqrt{N_{i}\cdot N_{j}}}$$

Here, *N*_*i*_ and *N*_*j*_ are the number of heavy atoms of residues *i* and *j*, respectively. *N*_*ij*_ is the number of heavy atom contacts of *i*, *j* pairs within a given cutoff distance of 4.5 Å. This cutoff value is commonly used^39,40^ since it effectively captures noncovalent interactions (hydrogen bonds, van der Waals contacts, and hydrophobic interactions). The inverse of *a*_*ij*_ is assigned as the weight of the edge between the *i* ^th^ and *j* ^th^ residues following the power law, assuming that residue pairs with a high number of heavy atom–atom contacts are “close” in the network.^29^ This weighting suppresses the strong bias for the covalently bonded interactions and includes nonbonded interactions critical for the topology.

Three important centrality measures, betweenness, closeness, and degree, reveal the properties of the nodes in the network, and these properties can be associated with functional roles of the residues in a protein structure.^29,31,32,34,36,41–43^ Betweenness centrality *C*_B_ measures how frequently a node appears on the shortest paths between other nodes in the network and is calculated as defined in eq 2,

2 $$C_{B,i}=\underset{s,t\in \mathrm{Paths}\left(V\right)}{\sum }\frac{\sigma \left(s,t|i\right)}{\sigma \left(s,t\right)}$$

where *C*_B,*i*_ is the betweenness of the *i* ^th^ node, *V* is the set of all nodes, σ­(*s*, *t*) is the number of shortest paths between nodes *s* and *t* and σ­(*s*, *t*|*i*) is the number of such paths that involve node *i*. The nodes with the highest betweenness values referred as “hub residues”, have a high capacity in receiving and distributing information within the protein network.^29,31,34,41^ Closeness centrality value *C*_C,*i*_ for the *i* ^th^ node shows its average farness to the other nodes and is defined as follows eq 3:

3 $$C_{C,i}=\frac{\left(n-1\right)}{\underset{k\neq i}{\sum }d\left(k,i\right)}$$

where *d*(*k*, *i*) is the shortest path distance between residues *i* and *k*, and *n* is the total number of reachable residues in the given network. Degree centrality, *C*_D,*i*_ is the number of edges connected to *i* ^th^ node (or number of neighbors), and is thus a local measure.

The adjacency matrix **A** containing 1/*a*_*ij*_, the inverse of the local interaction strength between *i* ^th^ and *j* ^th^ residues, is used to compute the graph Laplacian **L** = **D** – **A**, where **D** is the diagonal matrix of node degrees. The Laplacian matrix is decomposed to obtain its eigenvalues and eigenvectors. The eigenvectors are sorted according to their eigenvalues from smallest to the highest. The Fiedler vector corresponds to the smallest nonzero eigenvalue of **L**, and partitions the network into two subgraphs, where nodes are grouped according to their signs, either positive or negative. Similarly, the following eigenvectors partition the network into smaller subgraphs as the eigenvalue increases. The nodes at the edge of the dynamic domains constitute the hinge domains that coordinate the functional domain motions in protein structures,^44^ and indicate functional structural motifs.^45^ In line with this, nodes where a sign change occurs between consecutive positions are noted as hinge region residues,^46^ provided that both residues belong to the same chain. These initial assignments are then refined by comparing the absolute magnitudes of consecutive eigenvector values. If the magnitude decreases from residue *i* to *i* + 1 at a sign-change position, the hinge is shifted to *i* + 1, as this suggests the zero-crossing point lies closer to the neighboring residue, improving the accuracy of hinge detection. In this study, the Fiedler vector and the following two eigenvectors are selected for the hinge analysis. However, the number of eigenvectors can be adjusted by the user.

The RinPy package also performs a comparative analysis to reveal the differences between two networks—unperturbed and perturbed structures—to understand the effect of a perturbation on the contact topology of the protein complex, such as due to ligand binding or a mutation. In the analysis, two networks are not required to have the same size due to different numbers of amino acids and/or the presence of ligands since RinPy makes a direct comparison over the mutual amino acids. The betweenness, closeness, degree scores, and edge weights from the two networks are displayed as interactive scatter plots to show the increase/decrease in the corresponding node values.

In addition, a spectral community comparison between two networks is performed. For this aim, the first nonzero 20 Laplacian eigenvectors are computed for each network. To focus on community partitioning, values in each eigenvector set are transformed into sign representations (+1, −1). Then, the inner dot products between the modes of the two networks are calculated to determine partition overlap, as in our recent study.^33^

The hub residues have a high capacity to send/receive allosteric signals in the form of perturbations. As previously shown,^29,33^ the hub residues with high betweenness values are positioned such that they form a residue pathway linking distant functional regions of the protein. Thus, the positioning of the hub residues on the unperturbed and perturbed structures gives useful insights into how a perturbation may propagate in different states. In addition, graph spectral analysis is used to calculate the eigenvectors of the Laplacian matrix and investigate the eigenvalue shift percentages upon a perturbation at low eigenvalues, such as due to a ligand binding, as in a previous study^47^ using the following eq 4,

$$\%\;\mathrm{shift}\;\mathrm{for}\;\mathrm{mode}\;i=\frac{\lambda_{i,\mathrm{perturbed}}-\lambda_{i,\mathrm{unperturbed}}}{\lambda_{i,\mathrm{unperturbed}}}\times 100$$ 4

Here, λ is the eigenvalue for the *i* ^th^ nonzero mode of the perturbed and unperturbed structures.

The small value eigenvalues indicate the level of overall resilience of the network to perturbations. In terms of graph spectral analysis,^48^ when the *i* ^th^ eigenmode of two states (perturbed vs unperturbed) is compared, a larger algebraic value of λ_*i*_ suggests relative robustness of the network, while a lower value implies its vulnerability. Thus, a negative shift in a low-eigenvalue mode indicates a frustration in graph signaling that may inhibit the efficiency of flow of information, i.e., long-range allosteric communication dominated by low-frequency modes.

In the comparative analysis, we were also interested in allosteric communication between two distinct functional regions, marked by nodes *i* and *j*. We assumed that these node pairs are connected at least through one path—the shortest path—formed of *k* nodes. Assuming that the edges are undirected and are weighted based on their local interaction strengths, the efficiency of the path between nodes *i* and *j*, $E_{ij}^{\mathrm{path}}$ is calculated as defined in eq 5,^49,50^

5 $$E_{ij}^{\mathrm{path}}=\frac{1}{\overset{k-1}{\underset{l=1}{\sum }}w_{l,l+1}}$$

where *w*_*l*,*l*+1_ is the edge weight between two sequential nodes *l* and *l* + 1 on the path. We also calculated a sequential efficiency based on the average edge weight of the path as defined in eq 6,

6 $$E_{ij}^{\mathrm{seq}}=\frac{k-1}{\overset{k-1}{\underset{l=1}{\sum }}w_{l,l+1}}$$

To quantify the effect of ligand binding, an allosteric coupling score $C_{ij}^{\mathrm{path}}$ was defined based on $E_{ij}^{\mathrm{path}}$ , as the difference between perturbed and unperturbed states. Here, a negative value in $C_{ij}^{\mathrm{path}}$ indicates reduced communication of the *i* – *j* pair in the perturbed network.

### Workflow Overview RinPy

#### RinPy Package and Graphical User Interface

In this study, a Python-based open-source RinPy package was developed to create a RIN based on the contact topology of a protein structure and perform analyses on this network. The package determines centrality measures closeness, degree and betweenness of the nodes, identifies hub residues with a high capacity to receive and send a perturbation (such as due to a ligand binding), and decomposes the network into close-neighboring node domains (Figure 1). A graphical user interface (GUI) was developed as a front-end to the RinPy package (Figure 2).

**Figure 1:**
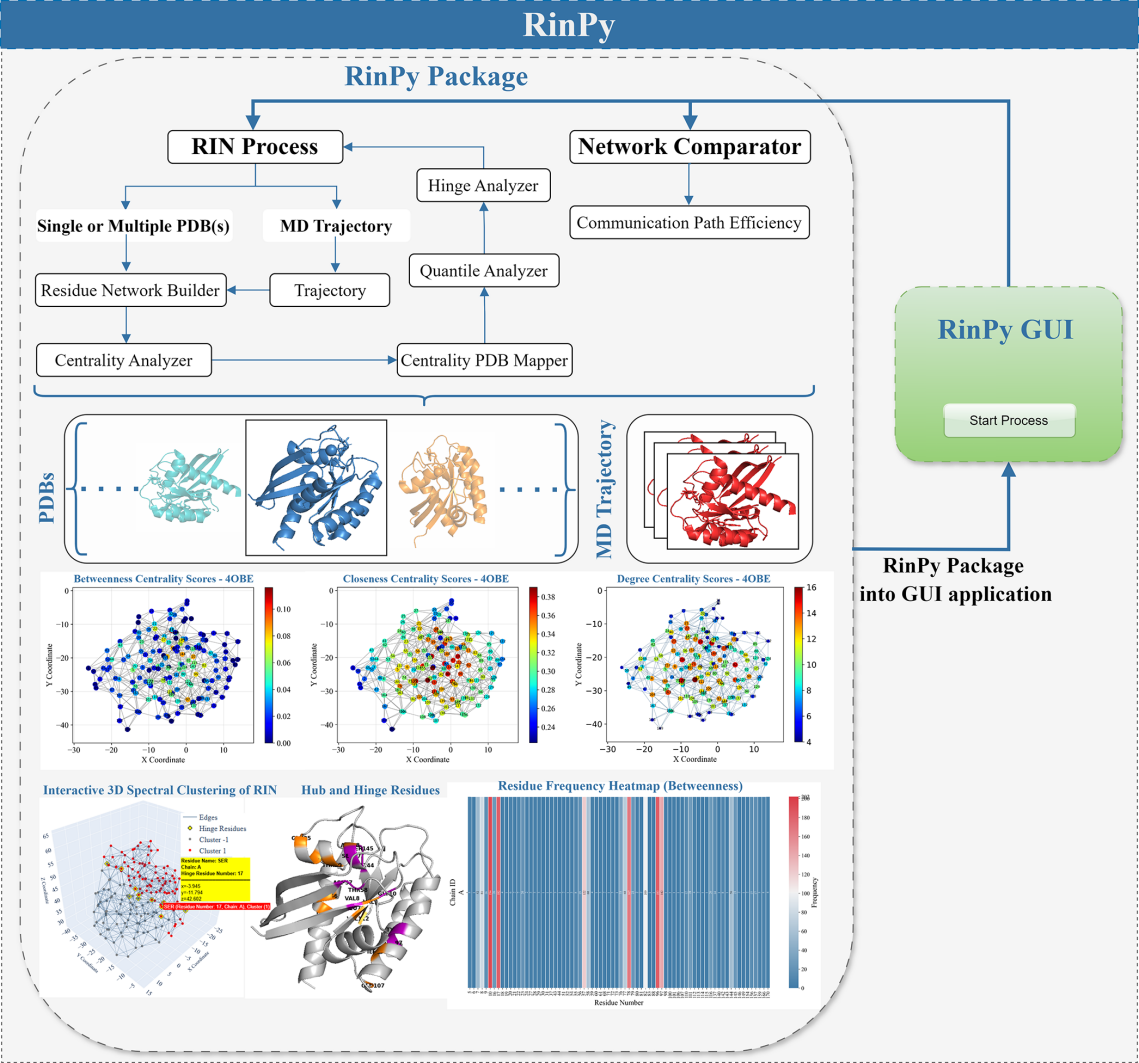
Flow of algorithms implemented in RinPy.

**Figure 2:**
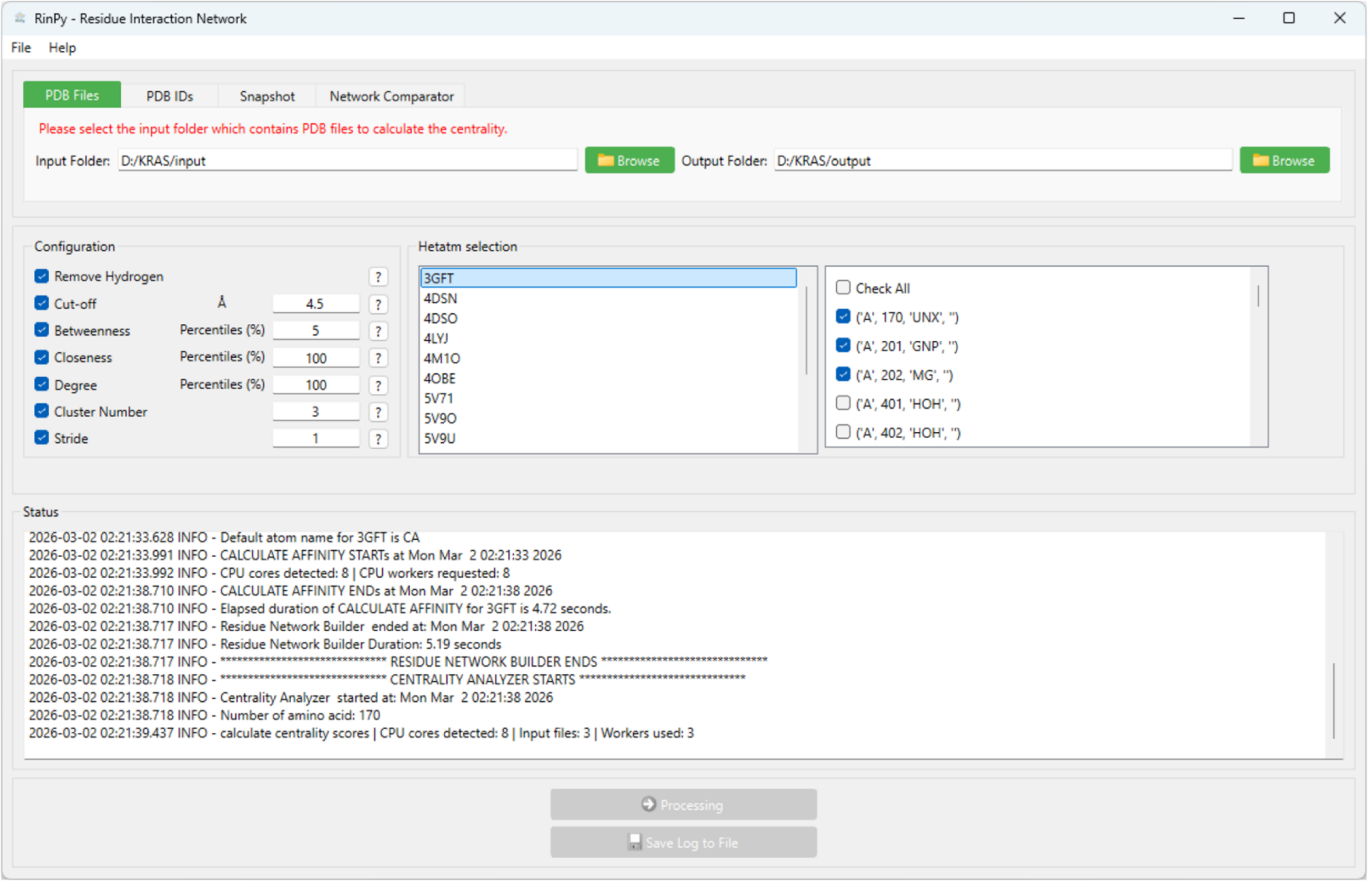
RinPy GUI interface is composed of three parts: input parameters, configuration, and status.

The RIN can be built using either experimental or simulation data (Figure 1). RinPy enables the submission of multiple data files simultaneously and the collection of outputs based on the structural analysis. Accordingly, the program accepts input in three ways: by selecting the directory containing PDB files, by entering PDB IDs to download from rcsb.org, or by using a single PDB file containing molecular dynamics (MD) snapshots. An output path must also be selected, and then, the program creates a directory under this output path with the corresponding PDB ID to save the results. For the MD trajectory, the stride parameter is exploited to split frames from a PDB that contains MD snapshots. This parameter allows skipping frames in the simulation trajectory for efficiency. For example, if the trajectory contains 1,000 frames, setting a stride value of 10 saves and processes every 10th frame as a PDB file, reducing the total number to 100 frames and significantly decreasing computation time. As a result, 100 folders will be created to save the results of each parsed frame with the name convention of 000x, such as 0001, 0011, and 0021, in the given output path.

Moreover, the program provides numerous options to the end-user, such as cutoff distance selection (default value is 4.5 Å), centrality type with quantile percentile (default values are 5% for betweenness, 100% for closeness and degree), whether to remove hydrogens, and to include heteroatoms in the network. All heteroatoms (ligands, cofactors, waters, and ions) can be included in the network as nodes to investigate their effects. In GUI, the heteroatoms to be included in the network are selected from the automatically opened heteroatom list for each PDB by navigating with the scroll bar. On the other hand, when the RinPy package is run from the terminal, the ligand dictionary JSON file, including selected heteroatoms, must be provided. Once all parameters have been configured, the user can initiate the calculation by clicking the ″Start Processing″ button from the GUI (Figure 2). The status panel on the interface provides real-time updates on the progress of computation with additional information such as calculation time. Additionally, users can save log details by clicking the ″Save Log to File″ button, and the program also automatically saves log details as a file into the selected output path. For each task, brief information is provided to the user next to the selection boxes.

### RinPy Backend Implementation

RinPy is designed as a Python package that can be shared on PyPI (Python Package Index), a platform for sharing and distributing Python packages. Users can install the package directly using the pip package manager. Also, the RinPy GUI is developed as a dependent on this package, designed to be user-friendly and available to the scientific community. In the RinPy package, the time-consuming process of computing heavy atom–atom distances is reduced through the use of a multiprocessing approach, thereby improving computational efficiency and reducing execution time. The number of processors available is automatically assigned to the calculations by the package.

The RinPy package comprises multiple modules (Figure 1), including the RIN process as a starter module, trajectory, residue network builder, centrality analyzer, centrality PDB mapper, quantile analyzer, hinge analyzer, network comparator, and communication path efficiency. Each module is designed to perform certain tasks independently of other modules. This design allows a new module to be easily integrated into this package later, if desired.

After the values for parameters are collected, the package initiates a full run for each input PDB. First, in the residue network builder, each residue is saved as the average of the Cartesian coordinates of its atoms and maintained as a single atom number in the residue average coordinate CSV file. The average coordinates of the residues and/or nucleotides are placed at the Cα atom or P atom, respectively, for protein-RNA/DNA complexes such as the ribosome and are saved as a PDB file. This module calculates the affinity between atoms of the residues in a multiprocessing way, which is then used to generate the RIN. In the pairwise affinity calculation, each residue in an *i*, *j* pair is first stored in a list as source and target residues, respectively. These residue pairs are subsequently distributed among the available processors in the running system. Each arbitrary processor takes source and target residues from the list, then calculates the Euclidean distance of the coordinates of their atoms to each other, and the number of those less than or equal to the given cutoff distance, such as the default value of 4.5 Å, is considered.

The centrality analyzer module takes the affinity values produced by the residue network builder module as input and constructs an interaction network consisting of nodes and edges using the NetworkX Python package.^51^ The weight of edges is calculated as the inverse of the affinity value between *i*, *j* residue pairs. Then, the centrality scores of betweenness, closeness, and degree are computed over the generated network in a multiprocessing approach. In the module, a threshold value is calculated by applying the given quantile percentile in the centrality scores, and those with values greater than or equal to this threshold are considered as high percentage residues for betweenness, closeness, and degree. PML files and PyMOL query strings are automatically generated to highlight these residues in the 3D protein structure in PyMOL.^52^

In the centrality PDB mapper module, the calculated centrality scores are replaced with the B-factor column of the PDB file, including the average coordinate of residue atoms, to generate centrality betweenness, closeness, and degree PDB files.

In the quantile analyzer module, the hub residues with high betweenness values calculated for each processed PDB file are aggregated to identify the common hub residues. For this purpose, a heatmap is generated to show the hub residues with their frequencies of occurrence, as an interactive HTML and PNG file. Similar to the betweenness centrality, the calculation can also be applied to closeness and degree.

In the hinge analyzer module, the Laplacian matrix is calculated and decomposed to obtain the eigenvalues and eigenvectors. For spectral analysis, the eigenvectors corresponding to the smallest nonzero eigenvalues based on the user-specified cluster number are extracted, including the Fiedler vector. The number of extracted eigenvectors is adjustable through a user-defined parameter referred to as the cluster number. Here, each eigenvector is referred to as a mode. In addition, each value in a mode is assigned a value of +1 or −1 based on the sign of its value, and these labels are subsequently mapped onto the B-factor column of the input PDB file, enabling visualization using spectrum coloring in molecular visualization tools. Additionally, a PML file for each mode is automatically generated to highlight hinge residues in PyMOL.

In the network comparator module, common nodes (residues) shared between two networks are identified, and differences in their centrality scores and edge weights are calculated. These differences are exported as a CSV file and visualized through a 2D PNG and an interactive 3D HTML file. For community structure analysis, the Laplacian matrix of each network is computed and eigen decomposition is performed to obtain their eigenvalues and eigenvectors based on a user-defined number of modes. The values in the eigenvectors are assigned −1, +1 based on their sign, and the inner dot product between the eigenvectors from two networks is calculated. The result is saved as a PNG heatmap. For graph signal processing, eigenvalue shifts are computed for the user-defined number of modes as the percentage change between the two networks eq 4. The resulting eigenvalue shift % plot is saved as a PNG file.

In the communication path efficiency module, efficiency values eq 5 and eq 6 of the shortest paths between user-defined source and target residues and the allosteric coupling scores are computed for two networks. The efficiency values of the shortest paths and allosteric coupling scores are exported as CSV files.

Table 1 shows the output files for each module in the RinPy package. Here, {PDB_ID} denotes the processed PDB ID; {Centrality_Type} is any of betweenness, closeness, or degree; {Mode_Number} is the number of selected eigenvectors and starts from 1 to *n*, where *n* is the adjustable user-defined parameter. Supporting Information includes the necessary details for the installation of RinPy, its features and examples.

**Table 1:**
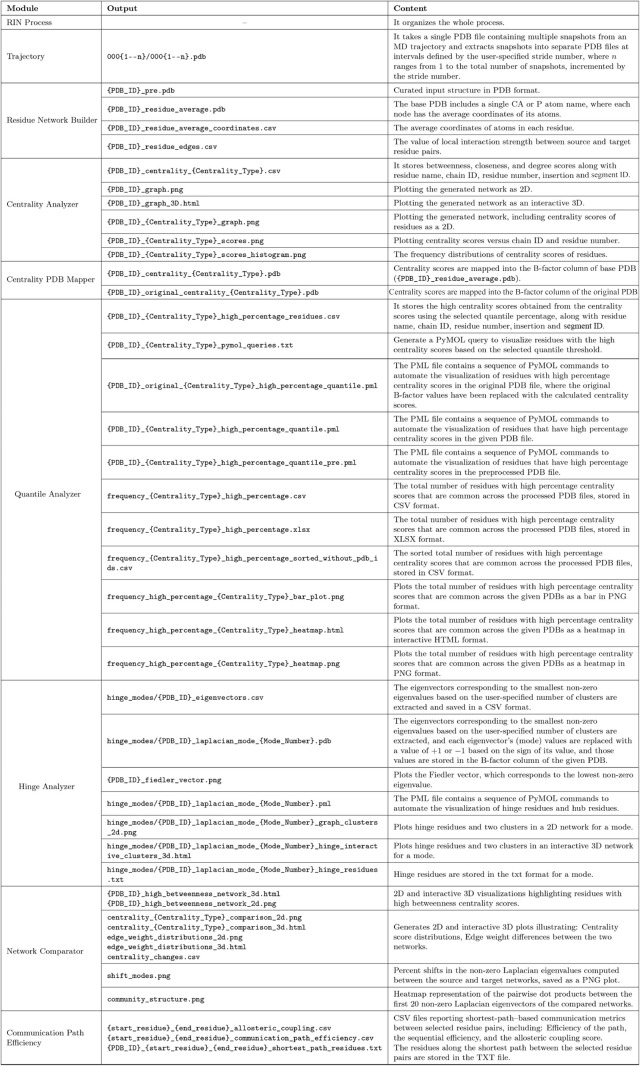
Output and Visualization Result Files Generated by Each Module in the RinPy Package

## Results and Discussion

### *Homo sapiens* KRAS Monomer and KRAS–SOS1 Complex

We implemented the RinPy program on a data set of human KRAS protein structures, encompassing both monomeric KRAS and KRAS–SOS1 complexes, as a case study. The purpose of this case study was to evaluate the method’s capacity to identify functionally significant allosteric sites across a range of KRAS conformational states. A total of 202 X-ray crystal structures of KRAS were obtained from the Protein Data Bank (rcsb.org), comprising wild type, mutants, apo forms, GDP- and GTP-bound states, and a total of 5 KRASG12C–SOS1 complexes (Table S1).

KRAS has three important functional sites, namely the P-loop (Gly10-Ser14), Switch-I (Asp30-Tyr40), and Switch-II (Thr58-Met72) to carry on the GTPase cycle.^53–56^ Each structure from the data set (Table S1) was subjected to RinPy, which calculated the centrality measures: betweenness, closeness, and degree of the residues. Here, Figures 3 and 4 show the box plots of betweenness, closeness, and degree centrality scores for the three functional sites on the investigated KRAS and KRAS–SOS1 complex structures, respectively. Betweenness of a node shows how frequently it is visited by the shortest pathways in the network. Closeness of a node indicates how close it is to the other nodes in the network, and the degree is the number of neighboring nodes (see section Theoretical Background). In the KRAS structures, the closeness values of the P-loop residues, acting as a nucleotide-binding site, are higher than those of Switch-I and Switch-II in all investigated KRAS structures (Figure 3). Active site residues generally have a high closeness value when compared to the rest of the structure, as closeness centrality reflects the effect of the whole protein on a single residue.^43^ Similarly, Switch-II residues Thr58 and Ala59 have high closeness scores, indicating that they have closer positions to other nodes. On the other hand, they demonstrate higher betweenness values than the P-loop, except for Gly10, and Switch-I regions. This finding suggests that these residues have a high capacity to receive and send information in the form of a perturbation in the structure, thus may have a role in allosteric communication or signal transduction. Moreover, Switch-II residues show a high degree of centrality, indicating higher local interaction in the network.

**Figure 3:**
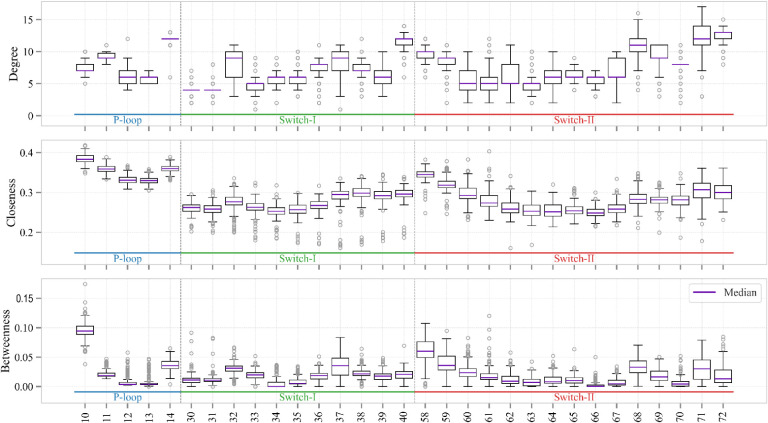
Box plot of betweenness, closeness, and degree scores of residues at the functional regions P-loop, Switch-I, and Switch-II, calculated for the investigated KRAS crystal structures.

**Figure 4:**
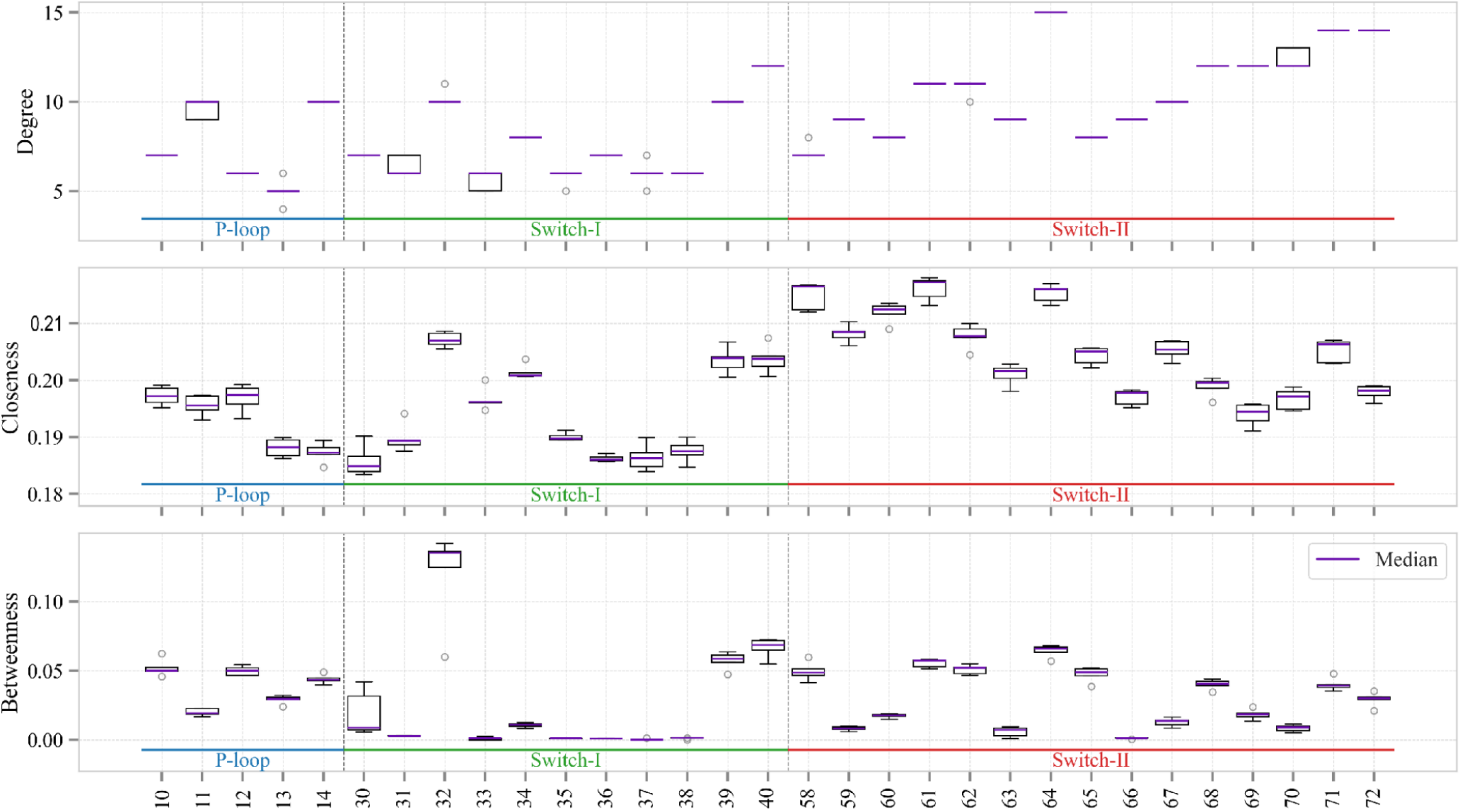
Box plot of betweenness, closeness, and degree scores of residues at the functional regions P-loop, Switch-I, and Switch-II, for the investigated KRAS–SOS1 crystal structures.

When comparing the centrality score distributions for the KRAS and KRAS–SOS1 functional sites, the closeness centrality scores of P-loop and Switch-I residues are higher in the KRAS monomer when compared to the KRAS–SOS1 structures. In contrast, the betweenness centrality scores of Tyr32, Ser39, and Thr40 on the Switch-I region residues are higher in KRAS–SOS1. The Switch-II residues also show higher degree centrality scores in the KRAS–SOS1 complex than those in the KRAS monomers (Figure 4). The Switch-I residues are noted to have more variable (GTP/GDP/conformation-dependent) closeness/degree scores compared to betweenness scores, whether in monomeric or complex form. To reveal any correlation between the centrality measures, we conducted an analysis using the Pearson correlation coefficients between centrality measures determined for the crystal structures in the data set. First, the centrality scores of residue *i*, *C*_B,*i*_, *C*_C,*i*_ and *C*_D,*i*_, calculated over all monomeric KRAS structures, are collected as arrays. Similarly, arrays are formed for each residue of the KRAS–SOS1 complex (5 different structures). Then, Pearson correlation coefficients are calculated between centrality measures for the residues in the functional regions. For each centrality measure, the centrality values of each residue within a given functional region across all PDB structures are concatenated into a single array. Pairwise correlations are calculated between different centrality measures using these arrays. As Figure 5 indicates for the functional regions of KRAS, Pearson correlation coefficients are high between betweenness and closeness values for P-loop residues (0.84) and Switch-II residues (0.74). The correlation is 0.50 for the Switch-I region, showing a different trend. While both measures refer to global centralities of the nodes, they have different implications. Closeness measures how fast an information will go from node *i* to other nodes; whereas betweenness measures the capability of node *i* to control the flow of information in the network. On the other hand, the correlations are relatively low for the betweenness and degree values of the P-loop (0.19), Switch-I (0.49), and Switch-II (0.47) regions implying that the number of neighbors does not necessarily lead to a capability to control flow of information. A similar finding was noted for the correlations between closeness and degree. These results are due to the contact topology of monomeric KRAS. The correlations for the KRAS–SOS1 complex show a different picture. Indeed, these results reflect the contact topology of KRAS in monomeric and complex forms. The Switch-I and Switch-II regions are located at the KRAS4B (one of the isoforms of KRAS)-SOS1 interaction interface. Thus, SOS1 engages the nucleotide-binding pocket of GDP-bound Ras through the insertion of an α-helical wedge, thereby displacing the Switch-I region from GDP and inducing conformational rearrangements in Switch-II that facilitate nucleotide release.^57^

**Figure 5:**
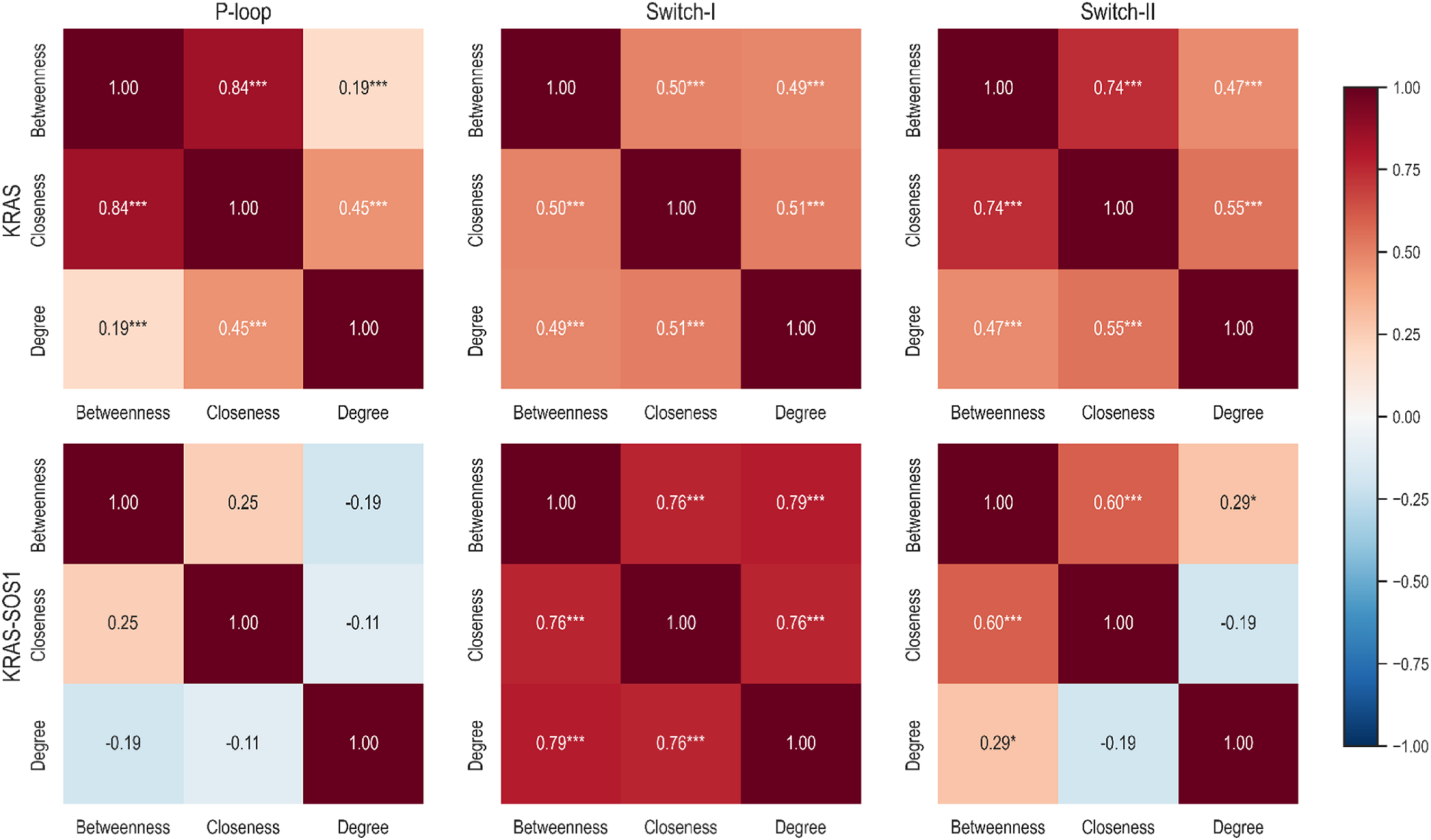
Pearson correlations between the centrality measures, calculated for the functional region residues of KRAS in monomeric form (upper panel) and KRAS–SOS1 in complex form (lower panel). *p < 0.05, **p < 0.01, ***p < 0.001.

We next focused on the residues with high betweenness scores that have a high capacity to mediate allosteric signaling pathways as they are common in the shortest paths throughout the network. Figure 6a and b show the residues with high betweenness scores (top 5% quantile, referred as hub residues) identified for the KRAS monomer and the frequency distribution of hub residues over a total of 202 KRAS monomer structures, respectively. The analysis of betweenness centrality reveals that the P-loop residue Gly10 and the P-loop neighbor Ser17 were consistently identified as hub residues with 96% and 90% frequency over 202 KRAS monomer crystal structures, respectively. A computational study using microsecond-scale molecular dynamics (MD) simulations^58^ has shown that Mg^2+^ formed a stable coordination bond interaction with Ser17, Asp57, and GDP, highlighting the importance of this region in the conformational change of KRASG12D relative to KRAS wild type and KRASG12C. Accordingly, in our study, Asp57, located next to Switch-II, frequently stood out with a high betweenness score in KRAS monomer structures. Similarly, the Switch-II region residue 58, observed as either Asp or Thr across the data set, was identified as a hub residue in 29% of these structures (Figure 6b). In the KRASG12D mutant, Val8 (near the P-loop)–Thr58 hydrogen bond interactions are markedly more persistent than in KRAS wild-type or KRASG12C.^58^ In line with this, sotorasib^59^ and adagrasib,^60^ which target a hidden allosteric pocket in the Switch-II pocket of the KRASG12C mutant,^61^ were approved by the FDA in 2021 and 2022, respectively.

**Figure 6:**
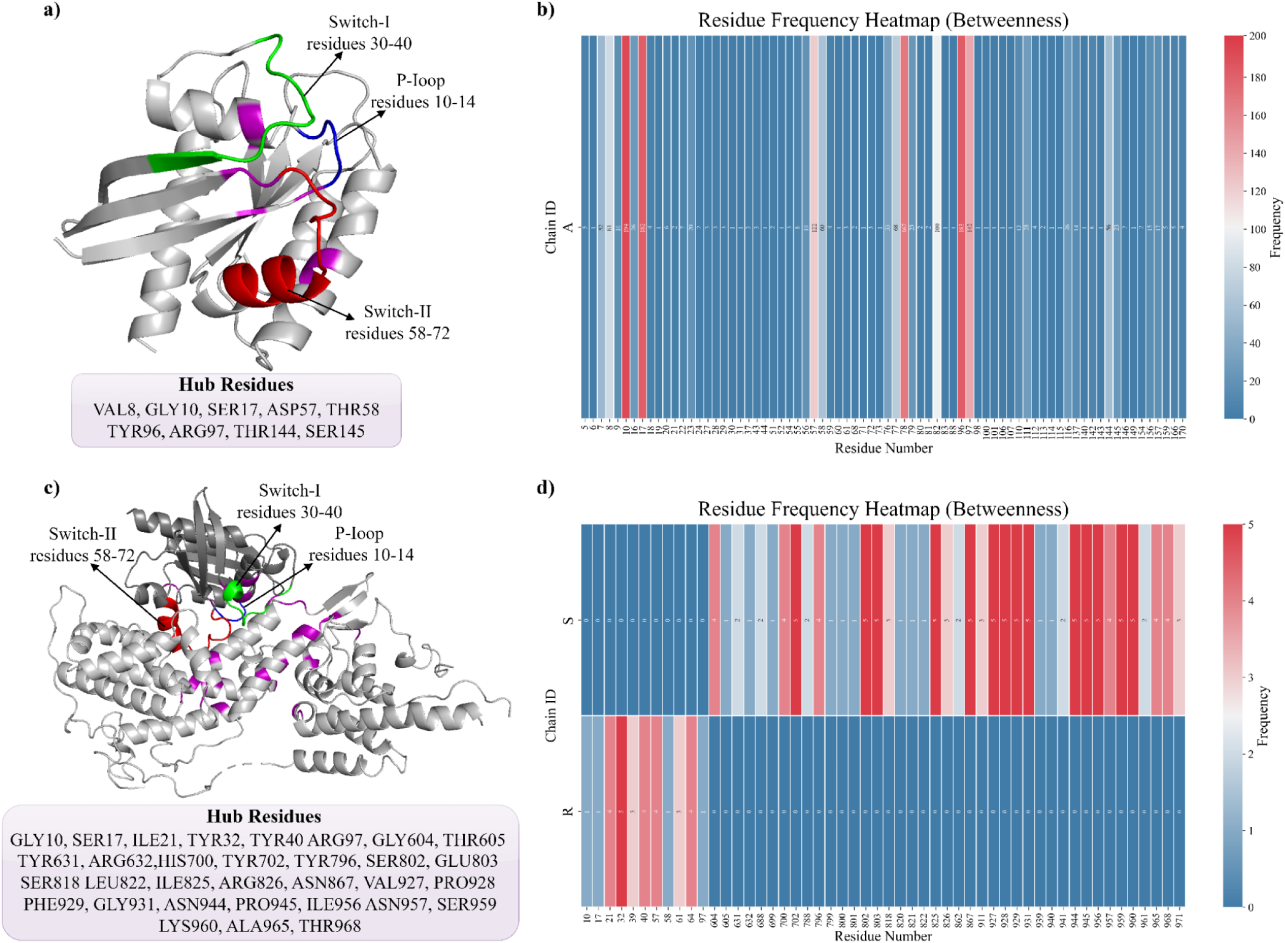
(a) The residues with high betweenness scores (hub residues) are colored in purple on the KRAS crystal structure (PDB ID: 4OBE), where the P-loop (in blue), Switch-I (in green), and Switch-II (in red) residues are also indicated. (b) The heat map for the frequency distribution of hub residues identified for a total of 202 *Homo sapiens* KRAS monomer structures. (c) The KRAS–SOS1 crystal structure (PDB ID: 6EPM) with the same coloring as in (a). (d) The heat map for the frequency distribution of hub residues identified for a total of 5 *Homo sapiens* KRAS–SOS1 complexes.

Computational analyses revealed multiple potential allosteric sites^62,63^ four of which (P1–P4) were later confirmed experimentally.^64^ Accordingly, in our study, Val7, Asp58/Thr58, Glu76, Gly78/Phe78 of P1, Gly10 and Tyr96 of P2, Glu76, Gly77/Glu77, Met111 of P3, Ser17 of P4 in KRAS and Ser39, Thr58 of P1, Gly10, Gln61 of P2, Ser17, Ile21, Tyr32, Ser39, Tyr40 of P4 in KRAS–SOS1 are predicted hub residues with high betweenness scores by RinPy. Agreeing with these results, a previous study using FTMap and accelerated MD simulations of KRAS reported eight hidden allosteric pockets, where 5 hit molecules were suggested for the P4 site.^65^

In line with the previously reported allosteric network involving residues Met72, Arg73, and Gly75 of the Switch-II region and Lys104 of KRAS,^66^ the RIN analysis revealed that Gly77/Glu77 and Gly78/Phe78 in spatial proximity to both Met72 and Gly75, were frequently identified as a potential allosteric residue across the majority of the 202 KRAS monomers. This suggests that Gly78/Phe78 may be functionally integrated into the established allosteric network of KRAS and highlights its potential role in modulating the oncogenic activity of KRAS mutants. Another notable finding of our current analysis is the recurrent identification of Tyr96 as a hub residue, which consistently exhibited a high betweenness centrality score in the KRAS monomers, highlighting its critical role in network communication. A previous study for KRASG12C/Y96D variant showed that the drug resistance mutation Y96D disrupts a key hydrogen bond between the hydroxyl group of Tyr96 and the pyrimidine ring of the inhibitor adagrasib (MRTX849), increasing the hydrophilicity of the Switch-II pocket and reducing the strength of chemical interactions between the drug and its target.^67,68^ In addition to the functional sites of KRAS, the allosteric P110 site^69^ residue Arg97/Tyr97 was projected to be a hub residue in 142 structures out of 202 KRAS structures examined in the data set (i.e., 70% frequency), indicating its functional significance. Indeed, Arg97 marks the P110 pocket where the inhibitor KAL-21404358 binds to KRASG12D, confirmed with NMR results.^69^

A previous study demonstrated that the KM12-AM nanobody binds to a cryptic pocket in KRAS,^70^ accommodating residues Leu23, Ile24, Gln43, Val44, Ile55, Arg149, Phe156, and Tyr157/Phe157 that emerged as hub residues in our RIN analysis with considerable occurrences. A recent computational study^71^ investigated the conformational dynamics of KRAS in complex with monobodies (12D1, 12D5) and affimer proteins (K6, K3, K69) and identified His95/Tyr96, Phe78/Leu80/Phe82, Val114/Asn116, and Tyr157/Leu159/Arg164 as universal hotspot residues in KRAS. Notably, RIN analysis also identified Tyr96, Gly78/Phe78, Phe82, Val114, Asn116, Phe157/Tyr157, and Leu159, as hub residues, supporting their key roles in KRAS allostery.

When KRAS binds to the SOS1, the contact topology changes, particularly at the protein–protein interface. Based on the investigated KRAS–SOS1 crystal structures, P-loop residues Gly10 and Ser17 were identified as hub residues with 20% and 20% frequencies, respectively (Figure 6d). As the Switch regions of KRAS form the binding interface for effector proteins, as well as for RAS regulators (GAPs and GEFs), in the KRAS–SOS1 complexes, these regions emerged as putative allosteric sites for the complex (Figure 6c and d). The Switch-I residues Tyr32, Ser39, Tyr40, and Switch-II residues Gln61 and Tyr64 were identified as hub with high frequencies. In addition, Asp57 located next to Switch-II stood out with a high betweenness score in the complex structures. Notably, Tyr32 plays a key role in regulating the conformational dynamics of the Ras GTPase cycle.^72,73^ In particular, SOS inserts a helix from its CDC25 domain into the RAS nucleotide-binding pocket, thereby opening the two switch regions and destabilizing nucleotide binding. In this interface, Tyr32 directly interacts with SOS Asn944, and the Tyr32 hydroxyl forms a hydrogen bond with Ser39 in Switch-I and a water molecule, helping to stabilize the open conformation of RAS.^72^ Tyr64 inserts into a hydrophobic pocket within SOS, where it forms a hydrogen bond with Gly931.^74^ Identifying Tyr32, Tyr64, Gly931, and Asn944 in KRAS–SOS1 complexes as hub residues using the RinPy package highlights their cooperative role in modulating Ras activation. Their interaction exemplifies how interprotein contacts can serve as dynamic allosteric switches. These findings suggest that targeting such residues may offer a viable strategy for allosteric inhibition at the Ras–GEF interface.^75^ Furthermore, RinPy was evaluated for the MD simulation trajectory. A 400 ns MD simulation of the KRASG12C-SOS1 complex (PDB ID: 6EPM) was performed to generate a diverse conformational ensemble using the Desmond simulation package of Schrödinger.^76^ The native ligand (BQ5) was removed before the simulation to avoid ligand interactions. To generate multiple PDBs from the resulting MD trajectory, snapshots were extracted every 5 steps, yielding a total of 200 PDBs. For each snapshot PDB, RIN analysis was performed using the RinPy package. As a result, the Switch-II residues Thr58 and Gln61, along with Asp57 of KRAS in chain R and the SOS1 α-helix residues Phe929, Gly931, and Asn944, together with additional residues Gly604, His700, Tyr702, Ala798, Val799, Gln800, Phe862, Asn867, Val927, Pro928, Pro945, Ile956, and Asn957 in chain S, were identified as hub residues exhibiting consistently high betweenness scores with a 50% or higher frequency across the trajectory. This analysis enabled the identification of dynamically conserved hub residues. Our findings also suggest a critical function of residues Gly931 and Asn944 in directing the KRAS–SOS1 interaction.

### Hinge Residues of KRAS and KRAS–SOS1 Complex

The hinge residues were detected through graph spectral analysis of the RIN, where sign changes in the values of the eigenvectors. As the number of clusters increases (using more eigenvectors for partitioning), the protein structure is divided into many smaller subdomains. Determining the dynamic domains helps to understand the flexibility and functional dynamics of the protein^44^ that further support drug design.^30^ Figure 7 displays the results of the graph spectral analysis for KRAS and the KRAS–SOS1 complex. The results point to the orthosteric and allosteric binding sites, as well as global hinge sites, underscoring the critical function of these sites in regulating overall structural dynamics. The Fiedler vector (eigenvector 1) indicates Ser17 of the P-loop in the KRAS structure (Figure 7a, PDB ID: 4OBE) and Glu31 of Switch-I in the KRAS–SOS1 complex (Figure 7b, PDB ID: 6EPM), as hinge residues consistent with their functional role. In addition, the hinge residue Ser17 in the KRAS structure (PDB ID: 4OBE) was also identified as a hub residue, highlighting this region as a putative allosteric site. In our recent study,^31^ these hinge regions were also identified by essential site scanning analysis (ESSA)^77^ and RIN analysis of the KRASG13D.SOS1 complex, where a natural compound library was screened against two putative allosteric pockets at the hinge sites and potential hit compounds were proposed for further *in vitro*/*in vivo* studies.

**Figure 7:**
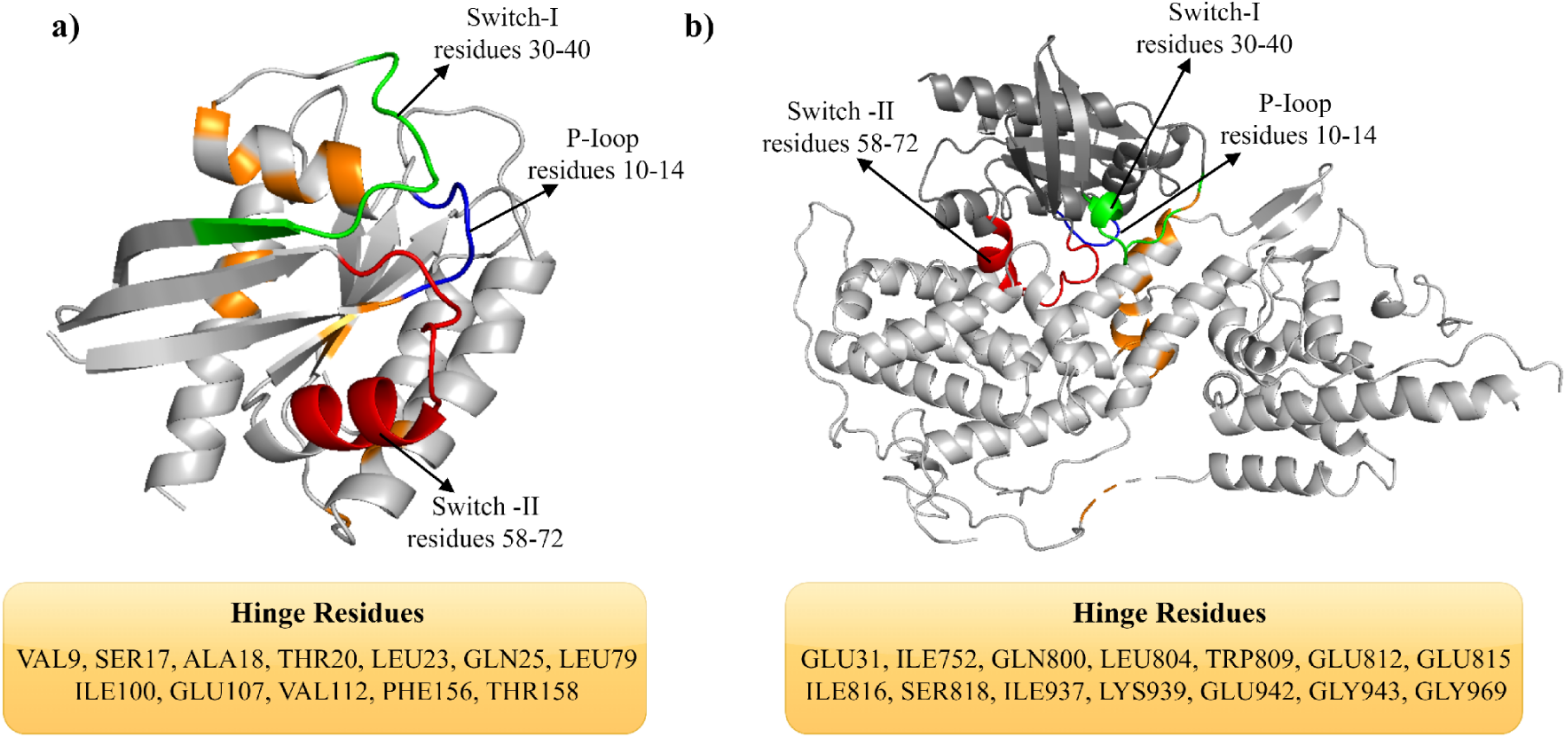
Hinge residues (in orange) determined from the Fiedler vector are shown for (a) the KRAS monomer (PDB ID: 4OBE) and (b) the KRAS–SOS1 complex (PDB ID: 6EPM). P-loop residues (10–14), Switch-I residues (30–40), and Switch-II residues (58–72) are indicated on the structures.

### Comparative Analysis between Two Different States of KRAS

RinPy enables the comparison of two different states, such as apo (PDB ID: 4OBE) and ligand-bound KRAS (PDB ID: 5V9U), where both structures also include GDP. It is worth mentioning that the structures are not required to have the same number of amino acids due to missing residues or the presence of ligands. Here, both KRAS structures have the same number of residues, which provides a direct comparison of the network metrics for each residue to showcase a ligand-binding-based perturbation example. RinPy was applied to ligand-free structures of KRAS to only consider the protein contact topology. Figure 8 illustrates the scatter plots showing the differences of the centrality scores, i.e., betweenness, degree and closeness, as well as the edge weights between those of the ligand-bound and the apo structure, where the latter represents the unperturbed state. RinPy generated interactive plots as.html files, which allowed navigating over the data to note the extent of changes in the centrality scores. Figure 8(a)-(c) indicates that the betweenness and degree of the residues considerably increased or decreased, while the closeness values showed a slight increase or decrease upon ligand binding. Focusing on the functional regions of KRAS revealed that the betweenness score of Arg68 (Switch-II residue) was highly decreased, implying a decrease in the capability of the residue to send/receive allosteric signals, whereas this measure was increased for Ala59 (Switch-II) upon ligand binding, suggesting an opposite effect. Ligand binding near Switch-II also changed the degree of the Switch-II residues. The most notable decrease in the degree score was noted for Glu62 upon ligand binding, due to the conformational change of Switch-II.

**Figure 8:**
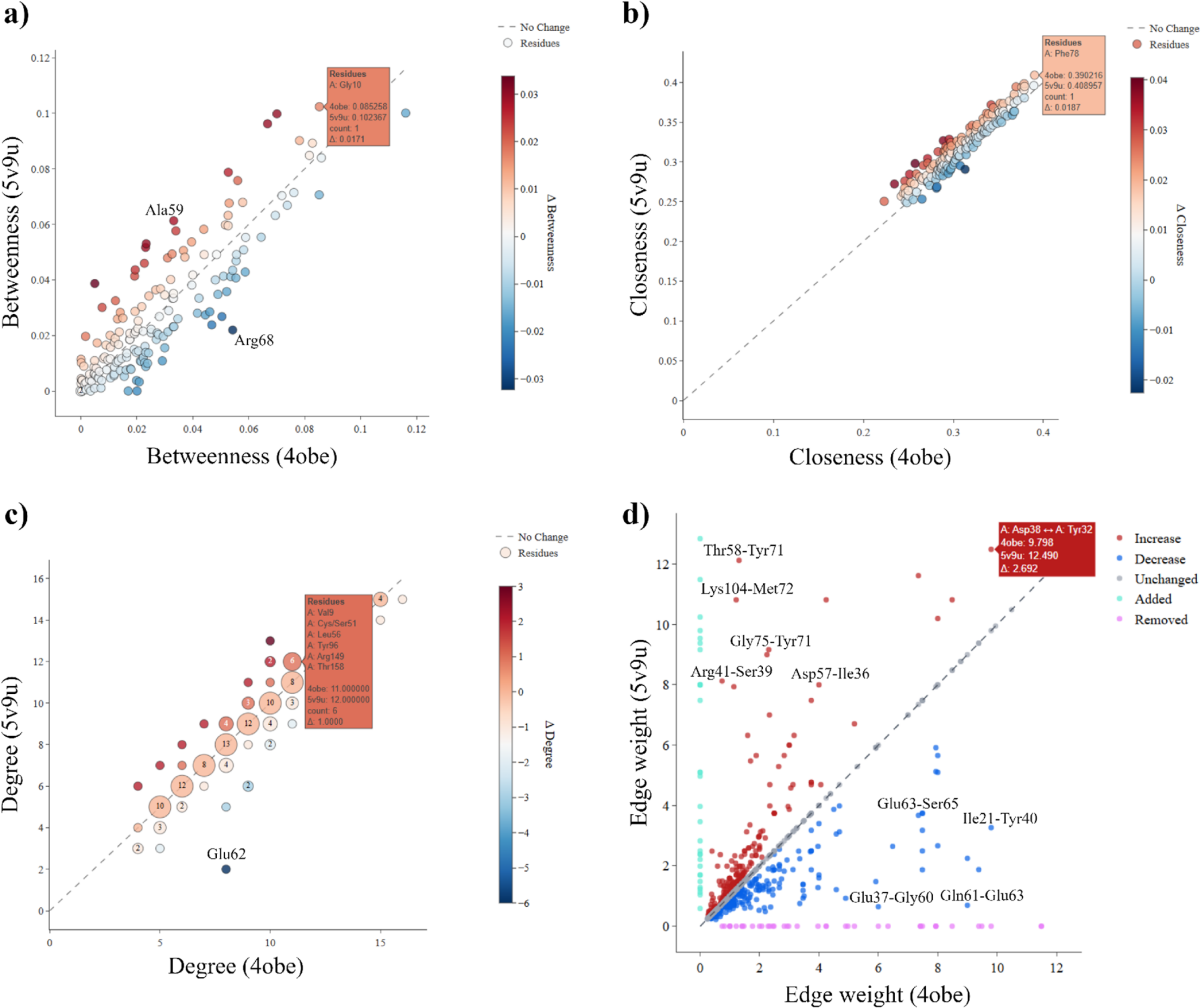
Comparative analysis between apo KRAS (PDB ID: 4OBE) and ligand-bound KRAS (PDB ID: 5V9U) to show the changes in (a) betweenness, (b) closeness, (c) degree values of the residues, and (d) edge weights between linked residues. The interactive scatter plots enable navigation over the changes in centrality measures for each residue and the edge weights in pop-up windows, where the color codes indicate high (red) to low (blue) values. In (c), the bubbles are sized according to how many residues share this value.

We also evaluated the differences in edge weights between the two structures (Figure 8(d)). Among the node pairs exhibiting increased edge weights in the ligand-bound structure, functional region residues, such as Ser39–Arg41 (both Switch-I), Ile36 (Switch-I)–Asp57 (Switch-II), Thr58–Tyr71 (both Switch-II), Tyr71 (Switch-II)–Gly75, and Met72 (Switch-II)–Lys104 pairs were noted. Conversely, decreased edge weights were observed for Ile21–Tyr40 (Switch-I), Glu37 (Switch-I)–Gly60 (Switch-II), Gln61–Glu63 (both Switch-II), and Glu63–Ser65 (both Switch-II). Taken together, these changes suggest that the ligand binding reorganizes the interaction network by strengthening certain residue pairs while weakening others, potentially altering the overall communication landscape of the protein.

Hub residues with high betweenness values have a high capacity to send/receive allosteric signals in the form of perturbations. As Figure 9(a) illustrates, the hub residues were positioned such that they formed a residue pathway linking distant regions of apo KRAS, i.e., the GDP binding site marked by Ser17 and a potential allosteric site indicated by Arg97 (Figure 9(a)). After ligand-binding, the majority of the hub residues were maintained, with new additions (Gln22, Leu23, and Phe78) (Figure 9(b)). The changes in the hub residues indicated the rewiring of the network after a perturbation, and how local changes may propagate in the structure.

**Figure 9:**
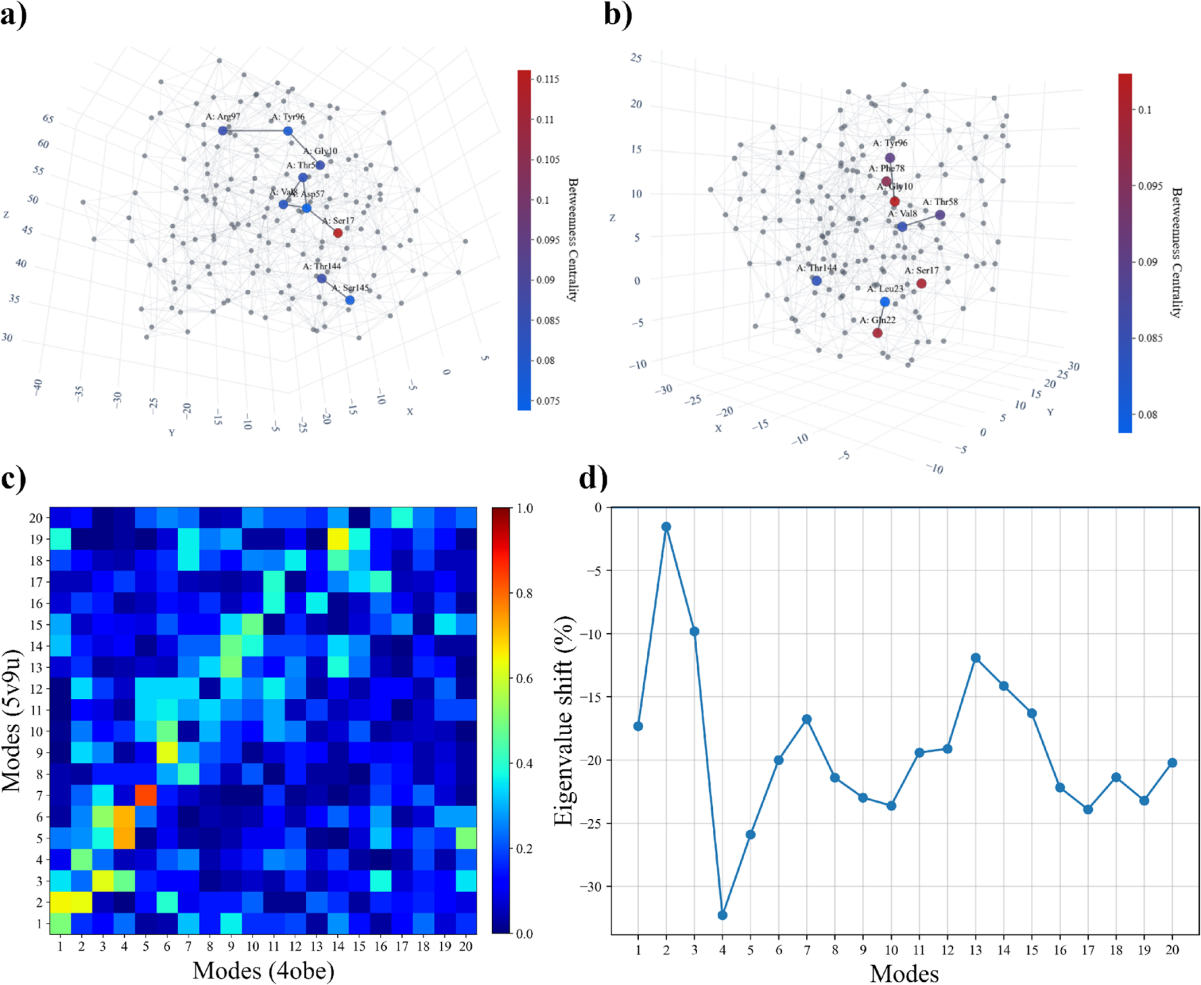
Hub residues with high betweenness values calculated for (a) apo KRAS (PDB ID: 4OBE) and (b) ligand-bound KRAS (PDB ID: 5V9U) are shown on the RinPy-generated plots as large spheres colored based on their centrality scores. (c) The heat map shows the inner dot products between the first 20 nonzero modes of the Laplacian matrix for the apo and ligand-bound states of KRAS. The dot products are between [0, +1] where the colors blue to red show increasing similarity between two community structures. (d) The eigenvalue shift (%) is plotted for the first 20 nonzero eigenvectors of the Laplacian matrices of apo and ligand-bound KRAS.

The community structures of apo and ligand-bound KRAS structures were compared. The inner dot products between the first nonzero 20 eigenvectors obtained from graph spectral analysis are displayed in Figure 9(c). The structural decomposition of two networks is highly similar, especially at the lowest-frequency modes (1–3) that exhibit relatively strong diagonal overlap (>0.65), indicating conservation of global domain organization. In subsequent modes, the overlap shifts off-diagonal, suggesting reordering of internal community structure in the ligand-bound state. The graph spectral analysis results for the apo KRAS (PDB ID: 4OBE) and ligand-bound KRAS (PDB ID: 5V9U) also indicated that the eigenvalues of apo KRAS shifted down to ∼−30%, due to ligand binding (Figure 9(d)), showing a vulnerability in the network at the low-frequency end of the spectrum that may affect the long-range allosteric communication.

To investigate long-range communication within the structure, we calculated two efficiency measures, $E_{ij}^{\mathrm{path}}$ and $E_{ij}^{\mathrm{seq}}$ for the shortest path between the P-loop residue Gly10 and the allosteric residue Thr144, for both apo KRAS (PDB ID: 4OBE) and ligand-bound KRAS (PDB ID: 5V9U) (Table 2). Notably, the decrease in $E_{ij}^{\mathrm{path}}$ and $E_{ij}^{\mathrm{seq}}$ indicates that the ligand binding disrupts signaling along the specific Gly10–Thr144 pathway rather than uniformly attenuating global connectivity. To quantify the effect of ligand binding, we defined an allosteric coupling score $C_{ij}^{\mathrm{path}}$ based on $E_{ij}^{\mathrm{path}}$ as the difference between ligand-bound and apo states. Here, a negative value in $C_{ij}^{\mathrm{path}}$ indicates reduced communication in the perturbed network. The resulting score for the case study was negative. Collectively, these results suggest that the ligand binding induces a substantial reorganization of internal structural communities and demonstrates ligand-induced weakening of long-range allosteric communication in KRAS between the allosteric site and the P-loop.

**Table 2:** Shortest Path Efficiency Values and Allosteric Coupling Score for Apo KRAS (PDB ID: 4OBE) and Ligand-Bound KRAS (PDB ID: 5V9U)

	Apo KRAS	Ligand-bound KRAS
*E*_*ij*_^path^	0.387	0.353
$E_{ij}^{\mathrm{seq}}$	1.549	1.414
$C_{ij}^{\mathrm{path}}$	–0.034	

### Computational Performance of RinPy

The performance of the RinPy package was evaluated on a Linux machine equipped with 40 CPU cores (2 × Intel Xeon Gold 6248R). The data sets consisting of 20, 40, 60, 80, 100, 120, 140, 160, 180, and 200 *Homo sapiens* KRAS monomer PDBs were generated, where each larger set contained all PDBs from the preceding subsets. For each data set, RinPy processes the PDB files contained within that data set sequentially. For each PDB, all residue pairs are generated and distributed across available CPU cores, where each core computes the atom–atom distances for its assigned pairs. Multiprocessing is also applied during the centrality metric computations for each PDB. Accordingly, runtime scales approximately linearly with the number of PDB files (Figure 10). RinPy efficiently uses all CPU cores and does not introduce noticeable delays or extra cost from parallel processing. Processing 20 PDBs corresponding to a total number of residues 3352 required approximately 4 min, whereas the full data set of 200 PDBs (33500 residues) was completed in ∼39 min.

**Figure 10:**
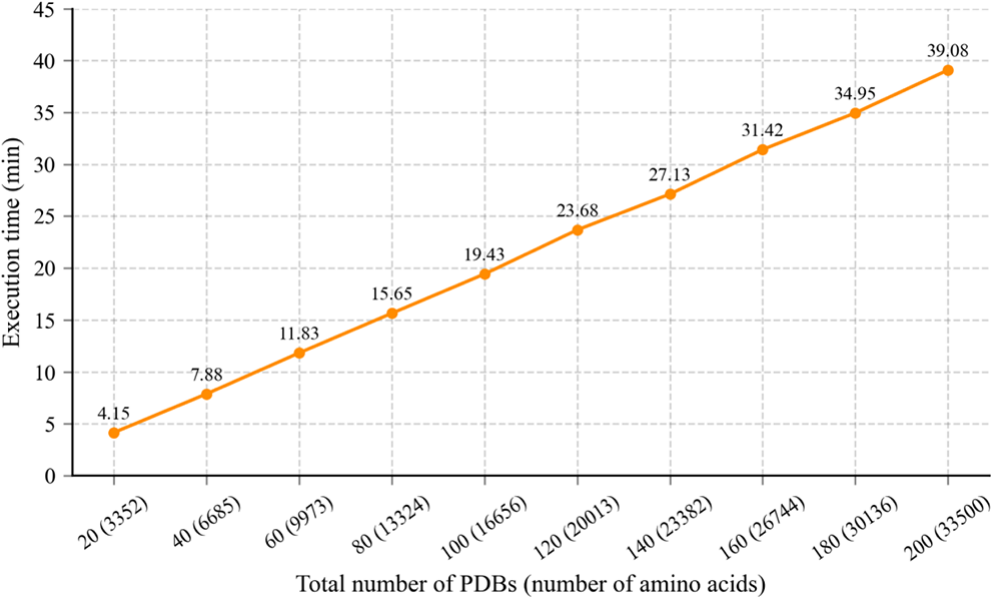
Processing time (minutes) plotted against the number of PDB files (number of amino acids) using RinPy with 40 CPU cores on 2 × Intel Xeon Gold 6248R.

## Conclusion

We present RinPy, a Python package with an accompanying graphical user interface designed for residue interaction network analysis of single or multiple PDB files as well as molecular dynamics trajectories. The contact topology-related network properties of the proteins may change according to their conformational dynamics. In line with this, RinPy integrates ensemble-scale statistical evaluation of multiple input structures and perturbation-aware comparative analysis within a unified graph theoretical framework. RinPy functions effectively as a standalone application and can also be seamlessly integrated into broader computational workflows. It enables the direct integration of nucleotides, cofactors, small molecules, water molecules, and ions as nodes within the interaction network, facilitating the interpretation of allosteric regulation. It also computes the frequency of hub residues with high betweenness scores based on multiple structural input PDB data and thus can identify conserved interaction motifs and functionally coordinated residue groups. The graph spectral analysis can be carried out for an adjustable number of eigenvalues to identify close-neighboring node clusters and the hinge regions that can coordinate global motions of proteins. Here, the hinge residues with high betweenness values are proposed as ligand-binding sites.^33,36^ Filtering out the hub residues with low solvent accessible surface area (SASA) is expected to highlight the potential allosteric pockets on the protein surface. However, this approach can miss the cryptic sites accommodating residues with low SASA that can be revealed during conformational sampling.

As a case study, the analysis of residue interaction networks of KRAS monomers and KRAS–SOS1 complexes was performed using RinPy. The results highly agree with previous findings on KRAS systems, thus demonstrate that RinPy provides valuable insights into functionally important residues that could be suggested for drug design and mutation studies. The RinPy package also performs a comparative analysis for two different states of the same protein, such as the unperturbed apo state of KRAS and the perturbed ligand-bound state of KRAS by focusing on differences in the centrality measures, edge weights, community structures, propagation of perturbation in the network, and efficiency metrics. This comparative analysis enables us to gain an in-depth perspective on allosteric mechanisms of protein structures based on their contact topologies and gives testable results with mutational studies. RinPy offers an easy-to-use platform for nonexperts new to the residue interaction network and allostery field, as well as experts who want to investigate network properties of protein structures within a unified and automated pipeline to collectively evaluate multiple input structures. The package currently supports betweenness, closeness, and degree centrality. Since the network is generated using NetworkX, any centrality metric provided by NetworkX (e.g., eigenvector centrality, PageRank) can be incorporated by extending the conditional blocks within the centrality analyzer module of the source code.

## Supplementary Material



## Data Availability

All data supporting the findings of this study are included in the article and its Supporting Information. The source code of RinPy is available on https://github.com/kurkcuoglulevitaslab/rinpy and https://github.com/zehrasarica/rinpy.

## Notes

Description of the RinPy package, including its software architecture, features, installation, and programmatic usage with examples; KRAS and KRAS–SOS1 structures analyzed in this study (Table S1) (PDF)

## Notes

For technical questions on RinPy, please contact Z.S. from zehraacar559@gmail.com and sarica16@itu.edu.tr.
