# Supplementary material for: RinPy, a Python Package for Residue Interaction Network Model to Analyze Protein Structures and Predict Ligand Binding Sites
Source: J Chem Inf Model. 2026 Apr 6;66(15):9507–22. doi: 10.1021/acs.jcim.6c00004 (PMC13471425; doi:10.1021/acs.jcim.6c00004)
Supplement: Supplementary file 1 [file ci6c00004_si_001.pdf]

SUPPORTING INFORMATION

RinPy, a Python Package for Residue Interaction Network Model to Analyze Protein Structures and Predict Ligand Binding Sites

Zehra Sarica[1], Fethiye Aylin Sungur*[1], and Ozge Kurkcuoglu*[2]

[1]Computational Science and Engineering Division, Informatics Institute, Istanbul Technical University, Istanbul 34469, Türkiye; sarica16@itu.edu.tr
[2]Department of Chemical Engineering, Istanbul Technical University, Istanbul 34469, Türkiye

*Email: aylin.sungur@itu.edu.tr; olevitas@itu.edu.tr

RinPy Package Description

This documentation provides a comprehensive overview of RinPy's architecture, features, installation, and programmatic usage with examples.

Software Architecture Overview

RinPy integrates ensemble-scale statistical evaluation of multiple input structures and perturbation-aware comparative analysis within a unified graph-theoretical framework. It enables systematic comparison between two states of a protein complex, focusing on perturbation propagation, community structure composition, allosteric coupling, and communication efficiency. RinPy provides two integrated core modules:

- `RINProcess`: construction of residue interaction networks and multiple analyses across single structures, large PDB ensembles, and PDBs from an MD trajectory, such as centrality analysis, detecting the residues with high centrality scores, frequency of common hub residues, and graph spectral analysis to detect potential hinges. It orchestrates sub-modules within a unified pipeline to perform the necessary analyses.

- `NetworkComparator`: performs a comparative analysis for two different states of the same protein, such as the unperturbed—apo or wild state—and the perturbed—ligand-bound or mutant state—by focusing on differences in the centrality measures, edge weights, community structures, propagation of perturbation in the network, communication path efficiency metrics, and allosteric coupling.

Features

RIN Process

- Conversion of protein complexes and protein-RNA/DNA complexes, such as ribosome, into residue interaction networks (RINs).

- Support for weighted edges based on local interaction strength or affinity.

- Node annotation with Chain ID, Residue Number, Insertion Code, Segment ID, and Cartesian coordinates.

- Scalable pipeline for single PDB structures, large PDB ensembles, and PDBs from an MD trajectory.

- Cross-platform compatibility (Windows, macOS, Linux).

- Export of analysis results in PyMOL-compatible formats (e.g., .pdb and .pml files) to enable direct visualization.

- Centrality analysis (betweenness, closeness, degree).

- Identification of high-centrality residues using user-defined quantile thresholds.

- Frequency analysis of common hub residues across PDB structures within the given dataset.

- Graph spectral analysis for detecting potential hinge residues and dynamically connected domains.

- Interactive 2D heatmap visualizations for centrality metrics, residue frequency distributions, and graph spectral analysis results.

- Interactive 3D visualizations of graph spectral analysis results mapped onto protein structures.

Network Comparator

Given a source and a target network (e.g., apo vs. holo), `Network Comparator` supports:

- Centrality comparison to quantify and visualize changes in degree, closeness, and betweenness centralities across the states.
- Edge weight comparison to detect increased, decreased, unchanged, added, and removed residue interactions between the two states.
- Community structure analysis to assess global domain organization and reordering of internal community under perturbation.
- Graph signal processing to evaluate perturbation-driven shifts in eigenvalue.
- Hub residue visualization to compare two states.
- Communication path efficiency analysis, such as sequential path efficiency, efficiency of the path, and allosteric coupling between user-defined residue pairs on the shortest path to quantify changes.
- Generation of comparative visual and tabular outputs, including 2D/3D network visualizations and structured CSV reports.

Installation

Requirements

- Python $\geq$ 3.10 and $<$ 3.14.
- RinPy depends on NetworkX $\geq$ 3.4, which requires Python 3.10 or newer.

We recommend using a Conda-based environment manager (Miniconda or Anaconda) to ensure stable dependency handling.

Installation via PyPI (Recommended)

RinPy is available on **PyPI** (rinpy) and can be installed directly using `pip`. The following steps demonstrate how to create and activate a conda virtual environment, install RinPy, verify the installation, and run the program from the command line:

```
# Create a conda virtual environment
conda create -n rinpy_env python=3.10 -y

# Activate the environment
conda activate rinpy_env or source activate rinpy_env

# Install RinPy
pip install rinpy

# Verify installation
rinpy --help
```

To run from the command line for the RIN process:

```
python -m rinpy --input_path INPUT_PATH \
      --output_path OUTPUT_PATH \
      --calculation_option_file path/to/calculation_options.json
```

or

```
rinpy --input_path INPUT_PATH \
      --output_path OUTPUT_PATH \
      --calculation_option_file path/to/calculation_options.json
```

To run from the command line for the network comparator:

```
python -m rinpy compare --source_input_path SOURCE_INPUT_PATH \
      --target_input_path TARGET_INPUT_PATH \
      --output_path OUTPUT_PATH \
      --residue_pairs "A:10;A:144" \
      --num_modes 20
```

or

```
rinpy compare --source_input_path SOURCE_INPUT_PATH \
      --target_input_path TARGET_INPUT_PATH \
      --output_path OUTPUT_PATH \
      --residue_pairs "A:10;A:144" \
      --num_modes 20
```

Parameter details for the terminal run for the RIN process:

- `--input_path`: Directory containing the input PDB files.
- `--output_path`: Directory where results and output files are saved.
- `--calculation_option_file`: JSON file containing configuration parameters. To download the example JSON file, click calculation_options.json.

Parameter details for the terminal run for the network comparator:

- `--source_input_path`: Directory containing the output of the RIN process, such as apo or wild states.
- `--target_input_path`: Directory containing the output of the RIN process, such as holo or mutant states.
- `--output_path`: Directory where results and output files are saved.
- `--residue_pairs`: Accepts a semicolon-separated list of residues in the format `chain_id:residue_number[:insertion][:segment_id]`, where `insertion` and `segment_id` should be provided only if available to ensure unique identification of the residue; otherwise, they may be omitted (e.g., `"A:10;A:144"` `"A:12;A:56"` or `"A:15:D:ABC;A:120:C:AB"`).
- `--num_modes`: Number of non-zero Laplacian eigenvalues used in community structure analysis. The trivial zero eigenvalue is excluded.

Installation from Source (Alternative)

If you prefer to install RinPy from source instead of using pip, follow the steps below.

Note: Replace `<username>` with the GitHub account hosting the RinPy repository.

```
git clone https://github.com/<username>/rinpy.git
# or
git clone git@github.com:<username>/rinpy.git

# If required (macOS/Linux):
chmod -R +x rinpy
cd rinpy
```

```
conda create -n rinpy_env python=3.10

conda activate rinpy_env
# or
source activate rinpy_env

pip install -e .
```

Alternatively, you may create a virtual environment using a supported Python version ($3.10 \leq$ **Python** < 3.14)**:**

```
# Example with Python 3.10
python3.10 -m venv rinpy_env

# Activate the virtual environment
# On Windows:
rinpy_env\Scripts\activate

# On macOS/Linux:
source rinpy_env/bin/activate
```

Programmatic Usage

RinPy can also be used programmatically through the `RINProcess` and `NetworkComparator` APIs. To perform comparative analysis, `RINProcess` must be executed first for all centrality types, as it generates the required input files for `NetworkComparator`, including centrality measures (betweenness, closeness, and degree) and the residue interaction network in GraphML format.

RINProcess Example

Create a Python file named **main.py**, insert the content given below, and execute the script via the terminal (python **main.py**) or an equivalent environment.

```
from multiprocessing import freeze_support
from rinpy import RINProcess

def main():

    calculation_options = {
        'betweenness': {'is_checked': True, 'value': 5},
        'closeness': {'is_checked': True, 'value': 100},
        'degree': {'is_checked': True, 'value': 100},
        'cluster_number': {'is_checked': True, 'value': 4},
        'cutoff': {'is_checked': True, 'value': 4.5}
    }

    rin = RINProcess(
        input_path="path/to/input/files",
        output_path="path/to/output",
        pdb_ids=None,
        ligand_dict=None,
        calculation_options=calculation_options,
        trajectory_file=None,
        stride=1
```

```
    )

    rin.start_process()

if __name__ == "__main__":
    freeze_support()
    main()
```

NetworkComparator Example

Create a Python file named **main.py**, insert the content given below, and execute the script via the terminal (python **main.py**) or an equivalent environment.

```
from rinpy import NetworkComparator

# Each residue is defined as a tuple in the format (chain_id, residue_number, insertion, segment_id).
# If insertion or segment ID is not available, use an empty string ''.
# The residue pairs are a list of user-defined pairs. Each residue must be specified as
# (chain_id, residue_number, insertion, segment_id).

comparator = NetworkComparator(
    source_input_path=r"path to apo",
    target_input_path=r"path to holo",
    output_path=r"path to result folder",
    residue_pairs=[(('A', 10, '', ''), ('A', 144, '', ''))],
)

# num_modes specifies the number of non-zero Laplacian eigenvalues used in community structure
# analysis.

comparator.run(num_modes=20)
```

Notes (RIN Process Module)

- `output_path` is mandatory.
- Only one of the following inputs must be provided to `RINProcess`: `input_path`, `pdb_ids`, or `trajectory_file`.
- `stride` is applicable only when a trajectory file is provided. It determines the interval at which frames are extracted, meaning that PDB structures are generated every `stride` frames from the trajectory.

Notes (Network Comparator Module)

`RINProcess` must be executed first for all centrality types (betweenness, closeness, degree), as it generates the required input files for `NetworkComparator`.

Table S1: KRAS and KRAS-SOS1 structures used in the study.

System	State	Nucleotide	PDB ID(s)
KRAS	G12D	GSP	4DSO
KRAS	K104Q	GDP	6WS2
KRAS	A146T	GDP	8EDY
KRAS	A59G	GCP	6BP1
KRAS	A59G	GDP	6ASE
KRAS	A59E	GDP	7KMR
KRAS	D33E	GDP	6ASA
KRAS	G12A	GDP	5VP7, 5VQ0
KRAS	Q61A	GDP	5VQ1
KRAS	G12A	GTP	5VPI
KRAS	wild type	GTP	5VQ2
KRAS	G12A	GTP-gamma-S-GSP	5VPZ
KRAS	wild type	GTP-gamma-S-GSP	5VQ6
KRAS	G12A	GppNHp	5VPY
KRAS	G12C	-	4NMM, 5KYK
KRAS	G12C	GDP	4L8G, 4LDJ, 4LYJ, 4M1O, 4M1S, 4M1T, 4M1W, 4M1Y, 4M21, 4M22, 5F2E, 5V6S, 5V71, 5V9L, 5V9O, 5V9U, 5YXZ, 5YY1, 6ARK, 6B0V, 6B0Y, 6N2J, 6N2K, 6OIM, 6P8W, 6P8X, 6P8Y, 6P8Z, 6PGO, 6PGP, 6T5B, 6T5U, 6T5V, 6TAM, 6TAN, 6USX, 6USZ, 6UT0, 7A1W, 7A1X, 7A1Y, 7O70, 7O83, 7OO7, 7R0M, 7R0N, 7R0Q, 7RP4, 7YCC, 7YCE, 8AFB, 8AFC, 8AQ5, 8AQ7, 8AZX, 8B6I, 8B78, 8DNI, 8DNJ, 8DNK, 8JGD, 8QUG, 8TVK, 8TXJ, 8TXK, 8TY2, 8TY8, 8TY9, 8V3A, 8X6R
KRAS	wild type	GDP	4LPK, 4OBE
KRAS	G12C	GDP	7A47
KRAS	G12C	GNP	8V39
KRAS	G12D	GCP	6GJ5, 6GJ6, 6GJ7, 6GJ8, 6QUU, 6ZLI, 8R7W, 8R7X, 4DSN, 4DST
KRAS	wild type	GCP-GDP	5UK9
KRAS	G12D	GCP-GDP	7T47

System	State	Nucleotide	PDB ID(s)
KRAS	G12D	GDP	5US4, 6ZL5, 7EW9, 7EYX, 7F0W, 7RPZ, 7RT1, 7RT2, 7RT3, 7RT4, 7RT5, 8AZY, 8JHL, 8TXE, 8TXG, 8TXH, 8WB1, 9BL0, 4DSU
KRAS	G12D-C118S	GDP	4EPR, 8FMJ, 8FMK, 8T4V
KRAS	C118S	GDP	4EPT, 4EPV, 4EPW, 8AZR, 8AZV, 8FMI, 9BAI, 9BAK, 5W22, 5VQ8
KRAS	C118S	GTP	5VQ2
KRAS	C118S	GSP	5VQ6
KRAS	G12V-C118S	GDP	4EPX, 4EPY
KRAS	G12V-S39C-C118S	GDP	4Q02, 4Q03
KRAS	G12D	GNP	5USJ, 6GOF, 5OCG
KRAS	G12D	GNP-GDP	7EWA, 7EWB
KRAS	G12D-K104Q	GDP	6WS4
KRAS	G12R	GDP	4QL3, 8CX5
KRAS	G12R	GMPPNP (GNP)	6CU6
KRAS	G12S	GDP	7TLE, 7TLG, 7TLK
KRAS	G12V	GDP	4TQ9, 5UQW, 7C40, 7U8H, 8AZZ
KRAS	G12V/D38P	GDP	5WHD
KRAS	G12V	GPPNHP (GNP)	6GOE
KRAS	G12V-S39C	GDP	8AFD
KRAS	G13C	-	7OK3, 7OK4
KRAS	G13D	GDP	4TQA, 6E6G, 8B00, 8ONV, 8UN3, 8UN4, 8UN5
KRAS	G13D	GNP	6E6F, 8EBZ
KRAS	K117N	GNP	8EIE
KRAS	M72C	GDP	5VBM
KRAS	P34R	GNP	6MTA, 6O36, 6O46
KRAS	Q61E	GCP	7LZ5
KRAS	Q61H	GNP	3GFT, 6GOM, 6GQX, 6GQY, 6FA2
KRAS	Q61H	GPPNHP (GNP)	6GQW, 6GQT

**System**	**State**	**Nucleotide**	**PDB ID(s)**
KRAS	Q61H	GTP	6MNX
KRAS	Q61L	GDP	4WA7
KRAS	Q61R	GNP	9O0S
KRAS	R151G	GDP	8T73
KRAS	T50I	GDP	7T1F
KRAS	V14I	GDP	6MQG, 6MQN
KRAS	wild type	GDP	6M9W, 6MBT, 6MBU, 6P0Z, 6PQ3, 8T71
KRAS	wild type	GMPPNP (GNP)	6MBQ, 6VC8
KRAS	wild type	GNP	8T72
KRAS	wild type	GPPNHP (GNP)	6GOD
KRAS	-	GDP	8ECR
KRAS	C118S	GDP	8AZR, 8AZV
KRAS-SOS1	G12C	-	6EPL, 6EPM, 6EPN, 6EPO, 6EPP
